# Supplementary material for: Recovery of Lysosomal Acidification and Autophagy Flux by Attapulgite Nanorods: Therapeutic Potential for Lysosomal Disorders
Source: Biomolecules. 2025 May 16;15(5):728. doi: 10.3390/biom15050728 (PMC12109497; doi:10.3390/biom15050728)
Supplement: Supplementary file 1 [file biomolecules-15-00728-s001.zip › biomolecules-3600617-supplementary.pdf]

Supporting Information for

Recovery of Lysosomal Acidification and Autophagy Flux by Attapulgite Nanorods: Therapeutic Potential for Lysosomal Disorders

Yuanjing Hao [1,#], Xinru Fan [1,#], Xiaodan Huang [1], Zhaoying Li [1], Zhiyuan Jing, Guilong Zhang [1], Yuxue Xu [1], Na Zhang [1,*] and Pengfei Wei [1,*]

[1] School of Pharmacy, Shandong Technology Innovation Center of Molecular Targeting and Intelligent Diagnosis and Treatment, Binzhou Medical University, Yantai, 264003, China
** Correspondence: zhangna20@bzmc.edu.cn (N. Zhang); pfwei@bzmc.edu.cn (P. Wei).*

Summary

Figure S1..Page S2

Figure S2..Page S2

Figure S3..Page S3

Figure S4..Page S4

Figure S5..Page S4

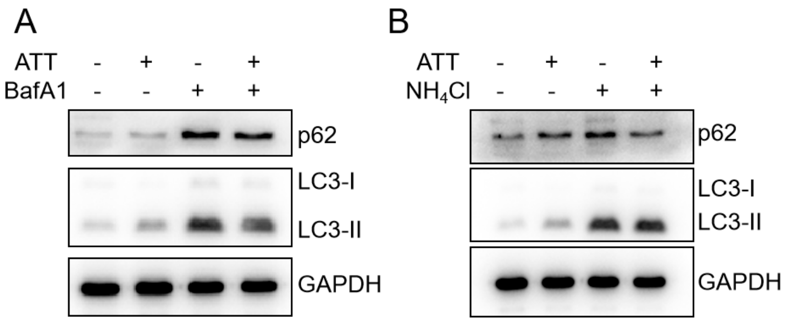

Figure S1 Restoring the degradation of autophagic substrates by ATT in Bafilomycin A1 and NH_4Cl treated HeLa cell. Immuno-blotting analysis of autophagic substrates expressions in HeLa cells with the indicated treatments for 24 h. (A) ATT (500 μg/mL), Bafliomycin A1(400 nM); (B) ATT (500 μg/mL), NH_4Cl (10 mM).

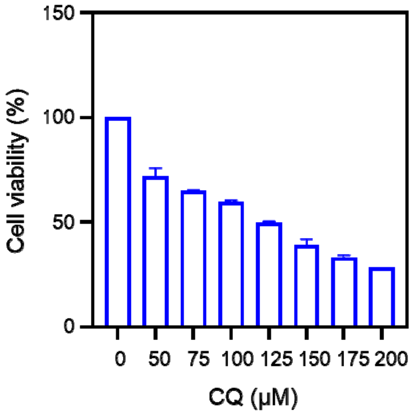

Figure S2 Cell viability of Hela cells via CCK8 assay. The cells were treated with different concentrations of CQ for 24 h. The experiments were performed twice.

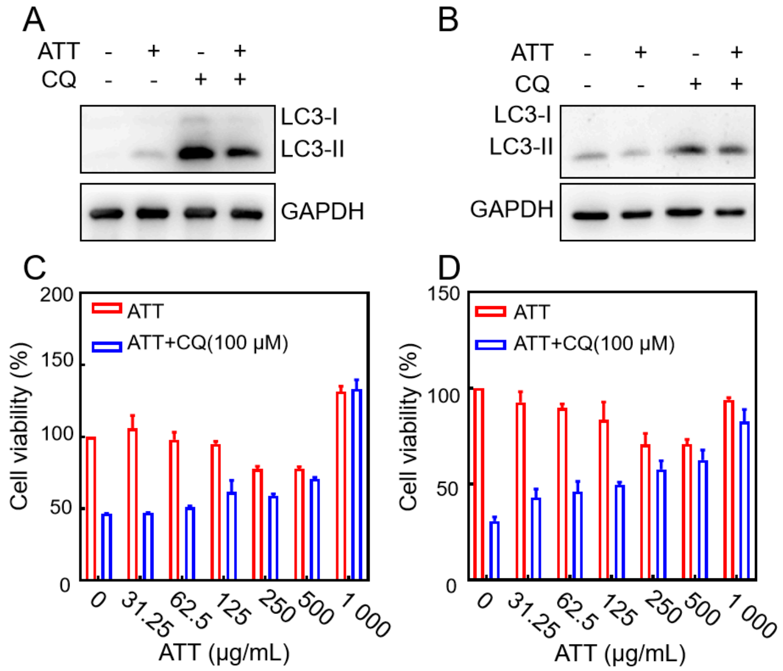

Figure S3 Immuno-blotting analysis of autophagic substrates expressions in (A) EA.hy926 cells and (B) HaCaT cells with the indicated treatments for 24 h. ATT (500 μg/mL), CQ (50 μM). Cell viability of (C) EA.hy926 cells and (D) HaCaT cells via CCK8 assay. The cells were treated with ATT (500 μg/mL) and CQ (50 μM) for 24 h.

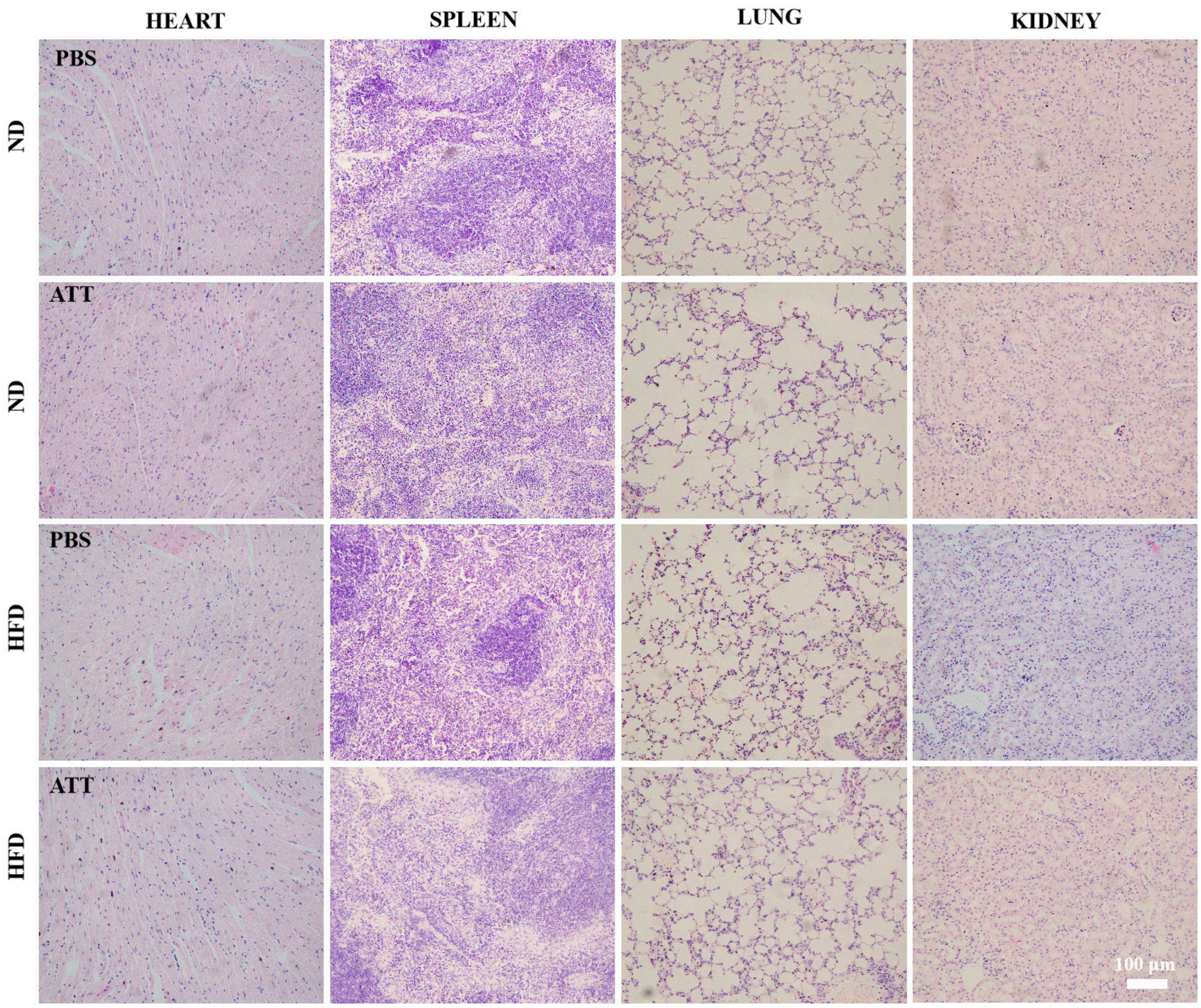

Figure S4 The effect of ATT nanorods on the histomorphology of major organs, including the heart, liver, spleen, lungs, and kidneys, in C57BL/6J mice. After the mice were fed either a normal diet (ND) or a high-fat diet (HFD) for 16 weeks, the histomorphology of the major organs of mice injected with PBS or ATT nanorods for 9 days was examined, respectively.

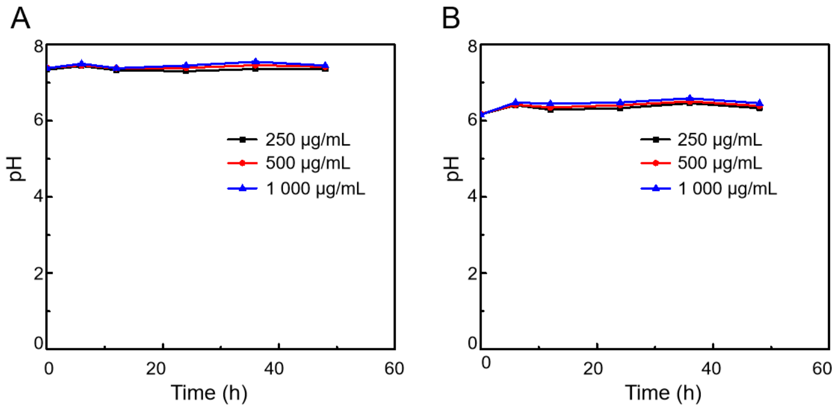

Figure S5 The pH-modulating capacity under either neutral (A) or mild-acidic conditions (B) mediated by different concentrations of ATT.
